# Perspective on Pulsed Electric Field Treatment in the Bio-based Industry

**DOI:** 10.3389/fbioe.2019.00265

**Published:** 2019-10-16

**Authors:** Leandro Buchmann, Alexander Mathys

**Affiliations:** Laboratory of Sustainable Food Processing, Department of Health Sciences and Technology, Institute of Food Nutrition and Health, IFNH, ETH Zurich, Zurich, Switzerland

**Keywords:** growth stimulation, continuous extraction, selective inactivation, pulsed electric field, bio-based industry

## Abstract

The bio-based industry is urged to find solutions to meet the demands of a growing world population. In this context, increased resource efficiency is a major goal. Pulsed electric field (PEF) processing is a promising technological solution. Conventional PEF and the emerging area of nanosecond PEF (nsPEF) have been shown to induce various biological effects, with nsPEF inducing pronounced intracellular effects, which could provide solutions for currently faced challenges. Based on the flexibility and continuous operation of PEF and nsPEF processing, the technology can be integrated into many existing cultivation systems; its modularity provides an approach for inducing specific effects. Depending on the treatment conditions, selective inactivation, continuous extraction without impeding cell viability, as well as the stimulation of cell growth and/or cellular compound stimulation are potential applications in the bio-based industry. However, continuous treatment currently involves heterogeneous energy inputs. Increasing the homogeneity of PEF and nsPEF processing by considering the flow and electric field heterogeneity may allow for more targeted effects on biological cells, further increasing the potential of the technology for bio-based applications. We provide an overview of existing and potential applications of PEF and nsPEF and suggest that theoretical and practical analyses of flow and electric field heterogeneity may provide a basis for obtaining more targeted effects on biological cells and for further increasing the bio-based applications of the technology, which thereby could become a key technology for circular economy approaches in the future.

## Introduction

Pulsed electric field (PEF) processing is a growing field in the area of electro-magnetic technologies for medical, environmental, and food applications (Toepfl et al., 2006b; Miklavčič et al., 2014; Postma et al., 2016; Raso et al., 2016; Buchmann et al., 2018b, 2019c). However, knowledge transfer and applications in bio-based industries (including yeast, lactobacilli, algae, and cell tissue production systems) have been limited. Raso et al. (2016) noted that the incomplete reporting of process protocols and insufficient characterization and control of pulse parameters need to be addressed to increase the implementation of PEF processing.

The treatment is based on the formation of a potential difference across a conductive biological material between two electrodes, creating an electric field that depends on the applied electric voltage, the shape of the electrodes, and the gap between electrodes, for further information on PEF parameter interconnectivity refer to Jaeger and Knorr (2017). PEF processing can be divided into conventional PEF processing in the range of micro- to milliseconds and nanosecond (nsPEF) processing (Beebe and Schoenbach, 2005; Mahnič-Kalamiza et al., 2014), in which high electric fields (10–100 kV cm^−1^) are applied for 1–300 ns. nsPEF induces intracellular effects, distinct from the pronounced effects of conventional PEF on the cell membrane (Kotnik and Miklavčič, 2006; Chopinet and Rols, 2015). Thereby, innovative applications and novel process windows are possible, while similar components for both treatments in batch and continuous mode are required (Toepfl, 2011; Buchmann et al., 2019c). In both cases, the resulting electropermeabilization increases the mass transfer of molecules and ions (Toepfl et al., 2006b). Depending on process parameters, a reversible or irreversible effect can be induced. Most current applications are focused on irreversible electropermeabilization, including non (minimal)-thermal pasteurization, enhanced drying rates, increased extraction yields, tissue softening as well as electrochemotherapy, and tumor ablation (Davalos et al., 2005; Toepfl et al., 2006a; Barba et al., 2015; Dermol et al., 2016; Golberg et al., 2016). Reversible electropermeabilization is typically used in molecular biology for the introduction of specific molecules, such as plasmids and antibodies, *in vivo* (Smith et al., 2004; Breton et al., 2012; Casciola and Tarek, 2016). However, the mechanisms underlying the PEF/ nsPEF induced effects are still the subject of intensive research (Teissie, 2017).

**Figure 1:**
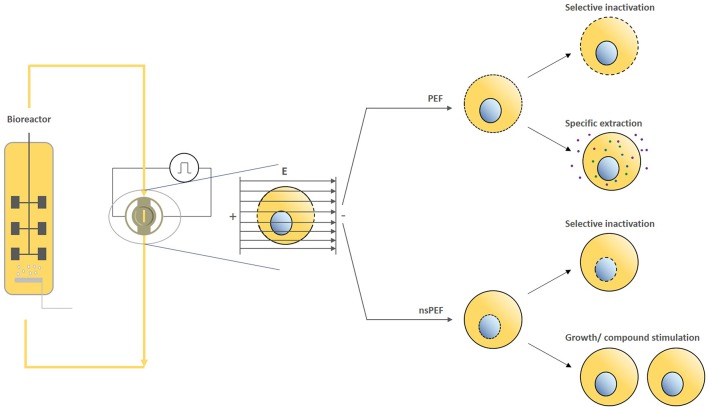
Exemplary working principle of PEF/nsPEF based processing of cultivated cells and their respective effects.

This perspective on PEF treatments in the bio-based industry summarizes basic principles of electropermeabilization by PEF/nsPEF and promising applications across different sectors (including targeted inactivation, the extraction of bioactive compounds, and the stimulation of cell growth and/or cellular compounds) (Figure 1). Furthermore, we note that increasing the homogeneity of energy input may lead to further improvements in efficiency and a wider array of applications and therefore is a key area for future research.

## Pulsed Electric Field Treatment in the Bio-based Industry

### Basic Principles of Pulsed Electric Field Processing

Scale-up approaches using nsPEF technology can benefit to a great extent from experience in the domain of conventional PEF processing (Buckow et al., 2010; Toepfl, 2011). However, PEF processing requires a multidisciplinary approach, including an understanding of innovative concepts within electrical engineering, fluid mechanics, and biology (Buchmann et al., 2018a,b, 2019c). The application of PEF to biological cells is based on the principle of electropermeabilization due to an induced transmembrane potential (Pauly and Schwan, 1959; Zimmermann et al., 1974; Schoenbach et al., 2004). The transmembrane potential difference as a function of time ΔΨ_*m*_*(t)* (V) can be derived from Equation (1) with form factor *f* (-) (1.5 for a spherical cell), electric field strength as a function of time *E(t)* (V m^−1^), cell radius *a*_*m*_ (m), angle with respect to the direction of the electric field θ (-), treatment time *t* (s), and membrane charging time τ_*m*_ (s), as defined in Equation (2) with membrane capacitance per unit area *C*_*m*_ (F) and extracellular σ_*e*_ and intracellular conductivity σ_*i*_ (S m^−1^).

(1) $$\begin{array}{l} \Delta {\text{Ψ}}_{m}\left(t\right)=f\cdot E\left(t\right)\cdot a_{m}\cdot \cos\theta \cdot \left(1-e^{-t/\tau_{m}}\right) \end{array}$$

(2) $$\begin{array}{l} \tau_{m}=a_{m}{\cdot C}_{m}\cdot \left(1/2\sigma_{e}+1/\sigma_{i}\right) \end{array}$$

To induce the required effect, the extracellular conductivity is of special interest. The membrane charging time (Equation 2) is strongly influenced by extracellular conductivity, as intracellular conductivity is fixed by the cell metabolism (Teissie et al., 2005). Additionally, extracellular conductivity needs to be in a range such that Equation (3) is equal to the pulse generator's resistance and hence matched load conditions are achieved (Küchler, 2009).

(3) $$\begin{array}{l} R=1/\sigma \cdot d/A \end{array}$$

where *R* is the resistance (Ω), σ is the media conductivity (S m^−1^), *d* is the electrode distance (m), and *A* is the electrode surface area (m^2^).

To assess the load for nsPEF, Equation (3) has to be extended, as shown in Equation (4).

(4) $$\begin{array}{l} Z_{tot}=1/\left(\sigma \cdot A/d+Y_{c}\right) \end{array}$$

where the total impedance *Z*_*tot*_ (Ω) is equal to the sum of the inverse resistance and the system's admittance *Y*_*c*_ (S) (Buchmann et al., 2018b).

For controlled PEF processing, the flow field distribution within chambers is an important parameter that has been neglected in energy input calculations to date (Meneses et al., 2011; Knoerzer et al., 2012; Raso et al., 2016; Buchmann et al., 2018a). The specific energy input *W*_*s*_ (J kg^−1^) can be calculated according to Equation (5), with pulse width τ_*p*_ (s) and number of pulses n (-),

(5) $$\begin{array}{l} W_{s}=\;E^{2}\cdot \tau_{p}\cdot \sigma \cdot n. \end{array}$$

The number of pulses can be derived from Equation (6), with frequency *f* (Hz) and residence or treatment time *t* (s),

(6) $$\begin{array}{l} n=f\cdot t \end{array}$$

From the author's perspective, the integration of the flow and electric field heterogeneities into the energy input calculation would facilitate the transferability of the results and the implementation of PEF on different scales and systems.

### Microbial Inactivation by PEF

The main advantage of PEF-based microbial inactivation is the ability to increase product quality while ensuring safety (Toepfl et al., 2006a; Mathys et al., 2013; Aganovic et al., 2017). PEF-based pasteurization has been widely investigated and industrialized and PEF-assisted sterilization has even been achieved under laboratory conditions (Toepfl et al., 2005; Raso et al., 2006; Reineke et al., 2015; Jaeger and Knorr, 2017). Although Aganovic et al. (2017) showed that PEF processing is actually more energy-intensive than thermal processing, its advantages could outweigh this current disadvantage, including its beneficial effects on quality due to lower thermal intensity, and therefore sustainability (Chaudhary et al., 2018; Chen et al., 2019) as well as its ability to selectively inactivate microorganisms. Nevertheless, the energy demand of PEF processing itself could actually be reduced by considering electric and flow field deviations in energy input calculations, as currently under- and over-processed areas appear simultaneously during PEF processing resulting in overall similar inactivation rates as compared to other techniques. A more homogeneous treatment, resulting from treatment chamber modifications and subsequent experimental planning, could help to overcome this limitation and even enhance the positive attributes of PEF processing.

Bio-based industrial cultivation relies on the use of specific microbial flora to ensure stable processes. However, the conditions and boundaries of industrial production commonly result in non-axenic and non-sterile cultivation to produce high value-added functional ingredients (including pharmaceuticals or biotechnological products), food, feed, and bioenergy. Therefore, a viable cell culture and thus stable cultivation system requires measures for microbial contamination control. For conventional PEF processing, predator control within a viable microalgae culture is possible, but not yet fully understood (Rego et al., 2015; Kempkes, 2016).

Moreover, selective inactivation has been achieved by nsPEF, allowing for reduced thermal effects and broader applications of the treatment owing to the greater similarity of organisms at the level of organelles than cell membranes (Buchmann et al., 2018b). However, selective inactivation is limited by two main factors. First, interactions of prokaryotic/eukaryotic consortia are not yet fully understood, resulting in unknown target organisms for selective inactivation. Second, the specific susceptibility of biological cells to electric fields has not been fully established, unlike in thermal processing (Kessler, 2002; Álvarez et al., 2006; Gianulis et al., 2017). However, since the entire processing principle is new, future developments in PEF/nsPEF-based culture stabilization by selective inactivation are anticipated. In addition, the concept of selective inactivation could be used to stabilize cultures after contamination, reducing bio-waste due to process failures. After the investigation of interactions in prokaryotic/eukaryotic consortia and their interdependence, specific process windows need to be established for targeted organisms in different growth phases with respect to environmental properties (pH, temperature, water activity, etc.). Despite current research on conventional PEF-based pasteurization, selective microbial control, which has enormous potential, should be a major focus of future PEF/nsPEF research.

### Extraction of Cellular Compounds by PEF Processing

PEF processing is suitable for biological applications that require gentle disintegration and extraction processes. The permeability induced by PEF processing results in increased mass transfer and thereby in higher extraction yields (Toepfl et al., 2006b; Bobinaite et al., 2015). Moreover, lower temperatures (e.g., 4°C) allow for the preservation of permeable structures without loss of cell integrity (Lopez et al., 1988). In addition, the selective nature of PEF-based extraction allows for the cascade processing of different cell-derived compounds such as carbohydrates, proteins, and lipids (Eing et al., 2013). However, PEF efficiency in terms of absolute yield and energy input is currently lower than those of other established processes (Postma et al., 2016; 't Lam et al., 2017). Two key parameters can explain the relatively low extraction yield. First, after PEF treatment, the permeable structure, which affects the cell membrane integrity, allows for the diffusion gradient assisted release of cellular compounds (Scherer et al., 2019). Second, PEF processing is able to permeabilize the cell membrane, but the lack of complete disruption, as obtained for example by bead milling, limits the extraction of membrane-bound compounds (Postma et al., 2016; Martínez et al., 2017).

Nevertheless, reversible PEF permeabilization is highly promising for selective microbial inactivation, and the concept of reversible and continuous PEF-based disintegration/extraction may have important future applications. This was demonstrated by Buchmann et al. (2019b), who showed that protein extraction without impeding growth is possible in *Chlorella vulgaris* cultures. In this system, protein extraction was highest after 24 h, resulting in a free protein extraction rate of 29.1 ± 1.1% and a *C. vulgaris* recovery rate of 93.8 ± 6.7% after 6 days. Regarding absolute yield PEF-based extraction yielded up to 96.6 ± 4.8% of the free protein fraction of *C. vulgaris*. However, high extraction yields were correlated with a reduced ability to grow after treatment in *C. vulgaris* cultures; hence, further research is necessary to identify optimal processing windows and to extend this approach to other taxonomic groups, such as yeast and bacteria.

Although initial studies have focused on proteins, permeability to various compounds, dependent on the media or solvent, should also be evaluated. Further research is necessary to identify the optimal processing window with regard to yield and growth rate. In-depth analyses of the cellular composition throughout the cultivation cycle in combination with treatment conditions favoring the extraction process are necessary. Moreover, the application of nsPEF to extraction processing could increase yields from organelle structures due to expanding on the efficiency for organelles.

The incorporation of membrane technology can potentially allow for the inline separation of targeted compounds and viable cells, thereby the continuous PEF-based extraction could enable circular economy concepts. Accordingly, cell engineering approaches for the excretion of targeted compounds might become obsolete. The integration of downstream processing in the upstream process could overcome current limitations in the bio-based industry, such as process heterogeneity, reproducibility, energy efficiency, and application portfolio.

### nsPEF Induced Growth Stimulation

Research on growth stimulation by an electric field has a long history; positive effects have been established in fungi, soy, microalgae, and other cells (Bertholon, 1783; Lemström, 1904; Bachman and Reichmanis, 1973; Takaki et al., 1984; Costanzo, 2008; Frey et al., 2011; Gusbeth et al., 2013; Mattar et al., 2015). However, controlled and reproducible growth stimulation was currently not possible. Electric fields and PEF may have precise stimulation windows, but controllable and reliable growth and/or compound stimulation has only been achieved under nsPEF conditions. Initial studies of growth stimulation based on nsPEF processing yielded promising results for *Arabidopsis thaliana* in a batch system and different laboratories (Eing et al., 2009; Songnuan and Kirawanich, 2012). In recent experiments, the transfer from a batch system to a continuous nsPEF process was successful, resulting in a 13.1 ± 1.6% increase in *Arthrospira platensis* SAG 21.99 biomass (Buchmann et al., 2019c). Moreover, an increase of 18.8 ± 5.5% and 19.5 ± 6% in allophycocyanin and C-phycocyanin, respectively, components of the economically important blue colorant phycocyanin, was obtained. Hence, nsPEF has the potential to increase growth as well as specific cellular compounds while maintaining techno-functional properties of the remaining compounds, as demonstrated for foaming, emulsification, and color compounds (Buchmann et al., 2019a,b).

Additionally, growth stimulation has been obtained in various organisms repeatedly treated with 100 ns pulses at 10 kV cm^−1^; photoautotrophic *Arthrospira platensis* SAG 21.99 (256 ± 67 J kg_sus_^−1^) (Buchmann et al., 2019c), photoautotrophic *Chlorella vulgaris* SAG 211-12 (360 ± 114 J kg_sus_^−1^) (Haberkorn et al., 2019), heterotrophic *Chlorella vulgaris* CCALA 256 (227 ± 60 J kg_sus_^−1^) (original data), and *Saccharomyces cerevisiae* DSM 70449 (173 ± 55 J kg_sus_^−1^) (original data) showed increased biomass concentrations after nsPEF processing of 13.1 ± 1.6%, 17.5 ± 10.5%, 12.2 ± 2.7%, and 20.5 ± 3.0%, respectively.

In this in-depth analysis, the pulse repetition frequency was adjusted according to the flow field. Under all investigated conditions, the increased growth was observed in a narrow processing window and required thorough process characterization and control (Buchmann et al., 2018a). Thereby, these effects could potentially be enhanced by a more homogeneous treatment, increasing the fraction of cells treated with the specifically required energy input.

**Figure 2:**
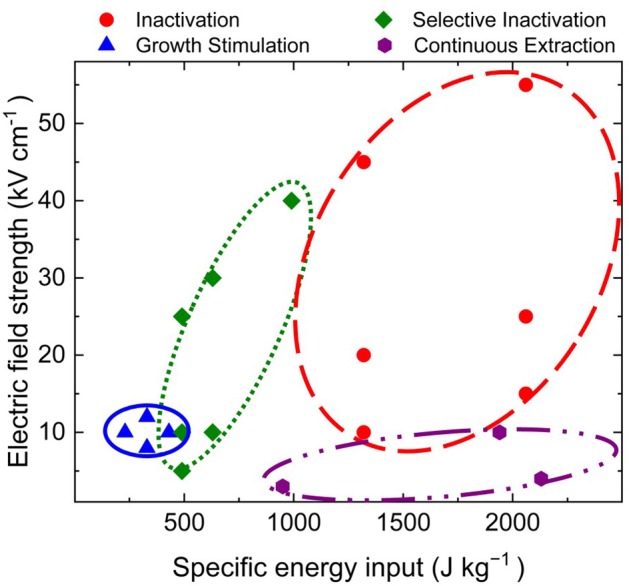
Case study of the microalgae *Chlorella vulgaris* SAG 211-12, illustrating treatment windows for selective inactivation, inactivation of microbial flora and *C. vulgaris*, continuous extraction of high value-added ingredients, and growth stimulation.

Moreover, successful treatment relied on the application of nsPEF at the early exponential growth phase, as shown by Buchmann et al. (2019c). These findings support the theory that the there is an increased effect of nsPEF on highly proliferating cells (Schoenbach et al., 1997). However, the specific mechanism underlying nsPEF-induced growth stimulation remains unknown. According to one hypothesis, it involves a Ca^2+^-based abiotic stress response pathway (Buchmann et al., 2019c). In addition to plants, fungi, and bacteria, the stimulative effect of nsPEF has been shown using animal cells and stem cells (Steuer et al., 2018; Ning et al., 2019). Ultimately, the effects on growth and parallel pigment production suggest that this technique can be used to enhance heterologous protein expression. Hence, nsPEF-based growth/cellular compound stimulation could be a viable strategy for future cultivation systems and may even be combined with continuous extraction or selective inactivation. Figure 2 summarizes a case study of the microalga *Chlorella vulgaris* SAG 211-12, illustrating treatment windows for selective inactivation, inactivation of microbial flora and *C. vulgaris*, continuous extraction of high value-added ingredients, and growth stimulation.

## Conclusions

Currently faced challenges in bio-based industries derived from a growing world population and simultaneously limited arable land require a change in current supply chains. The presented innovative concepts based on PEF/nsPEF processing bear the potential to be key processing steps toward more sustainable and efficient supply chains. In the domain of irreversible electropermeabilization, selective inactivation could enable inline microbial control, resulting in long-term stable cultivation and low contamination-related process failure. However, further studies of interactions between target cells and surrounding flora, and particularly on PEF/nsPEF-resistance of different strains, are needed for successful selective inactivation. The integration of downstream processing into upstream cultivation via the conventional PEF-based continuous extraction of specific cellular compounds without impeding cell growth can overcome current limitations, in bio-based industries, such as process heterogeneity, reproducibility, energy efficiency, and application portfolio. Moreover, nsPEF-based growth/cellular compound stimulation has the potential to increase resource efficiency, economic viability, and the affordability of the derived products, thereby meeting the demands of a growing world population. Given that PEF and nsPEF systems can be implemented in many existing cultivation systems via a bypass, it is also possible to combine the continuous extraction process with nsPEF-induced growth/cellular compound stimulation to enhance the overall performance of bio-based systems and ensure its long-term stability by selective inactivation.

Of note, continuous PEF processing in both domains is currently based on heterogeneous treatments conditions. Therefore, modified treatment chambers by the incorporation of flow and electric field distributions are necessary for more targeted and reproducible effects within cell cultures. Increasing the homogeneity of the treatment could further increase the induced effects of PEF/nsPEF. Thus, PEF and nsPEF have the potential to become high-impact technologies and to resolve current challenges in bio-based industries.

## Data Availability Statement

The datasets generated for this study are available on request to the corresponding author.

## Author Contributions

AM devised the main conceptual ideas. LB and AM discussed the ideas and commented on the manuscript. LB wrote the manuscript in consultation with AM.

### Conflict of Interest

The authors declare that the research was conducted in the absence of any commercial or financial relationships that could be construed as a potential conflict of interest.

## References

[B1] Aganovic K. Smetana S. Grauwet T. Toepfl S. Mathys A. Van Loey A. (2017). Pilot scale thermal and alternative pasteurization of tomato and watermelon juice: An energy comparison and life cycle assessment. J. Clean. Prod. 141, 514–525. 10.1016/j.jclepro.2016.09.015

[B2] Álvarez I. Condón S. Raso J. (2006). Microbial inactivation by pulsed electric fields, in Pulsed Electric Fields Technology for the Food Industry - Fundamentals and Applications, eds Raso J. Heinz V (Boston, MA: Springer, 97–129.

[B3] Bachman C. Reichmanis M. (1973). Some effects of high electrical fields on barley growth. Int. J. Biometeorol. 17, 253–262. 10.1007/BF01804618

[B4] Barba F. J. Parniakov O. Pereira S. A. Wiktor A. Grimi N. Boussetta N. (2015). Current applications and new opportunities for the use of pulsed electric fields in food science and industry. Food Res. Int. 77, 773–798. 10.1016/j.foodres.2015.09.015

[B5] Beebe S. J. Schoenbach K. H. (2005). Nanosecond pulsed electric fields: A new stimulus to activate intracellular signaling. J. Biomed. Biotechnol. 2005, 297–300. 10.1155/JBB.2005.297 16489262 PMC1361491

[B6] Bertholon P. (1783). De l'électricité des végétaux. Paris: Imprimerie de Didot Jeune.

[B7] Bobinaite R. Pataro G. Lamanauskas N. Šatkauskas S. Viškelis P. Ferrari G. (2015). Application of pulsed electric field in the production of juice and extraction of bioactive compounds from blueberry fruits and their by-products. J. Food Sci. Technol. 52, 5898–5905. 10.1007/s13197-014-1668-0 26345006 PMC4554608

[B8] Breton M. Delemotte L. Silve A. Mir L. M. Tarek M. (2012). Transport of siRNA through lipid membranes driven by nanosecond electric pulses: An experimental and computational study. J. Am. Chem. Soc. 134, 13938–13941. 10.1021/ja3052365 22880891

[B9] Buchmann L. Bertsch P. Böcker L. Krähenmann U. Fischer P. Mathys A. (2019a). Adsorption kinetics and foaming properties of soluble microalgae fractions at the air/water interface. Food Hydrocoll. 97:105182 10.1016/j.foodhyd.2019.105182

[B10] Buchmann L. Bloch R. Mathys A. (2018a). Comprehensive pulsed electric field (PEF) system analysis for microalgae processing. Bioresour. Technol. 265, 268–274. 10.1016/j.biortech.2018.06.010 29906715

[B11] Buchmann L. Böcker L. Frey W. Haberkorn I. Nyffeler M. Mathys A. (2018b). Energy input assessment for nanosecond pulsed electric field processing and its application in a case study with *Chlorella vulgaris*. Innov. Food Sci. Emerg. Technol. 47, 445–453. 10.1016/j.ifset.2018.04.013

[B12] Buchmann L. Brändle I. Haberkorn I. Hiestand M. Mathys A. (2019b). Pulsed electric field based cyclic protein extraction of microalgae towards closed-loop biorefinery concepts. Bioresour. Technol. 291:121870. 10.1016/j.biortech.2019.121870 31382092

[B13] Buchmann L. Frey W. Gusbeth C. Ravaynia P. S. Mathys A. (2019c). Effect of nanosecond pulsed electric field treatment on cell proliferation of microalgae. Bioresour. Technol. 271, 402–408. 10.1016/j.biortech.2018.09.124 30296747

[B14] Buckow R. Schroeder S. Berres P. Baumann P. Knoerzer K. (2010). Simulation and evaluation of pilot-scale pulsed electric field (PEF) processing. J. Food Eng. 101, 67–77. 10.1016/j.jfoodeng.2010.06.010

[B15] Casciola M. Tarek M. (2016). A molecular insight into the electro-transfer of small molecules through electropores driven by electric fields. Biochim. Biophys. Acta Biomembr. 1858, 2278–2289. 10.1016/j.bbamem.2016.03.022 27018309

[B16] Chaudhary A. Gustafson D. Mathys A. (2018). Multi-indicator sustainability assessment of global food systems. Nat. Commun. 9:848. 10.1038/s41467-018-03308-7 29487286 PMC5829192

[B17] Chen C. Chaudhary A. Mathys A. (2019). Dietary change scenarios and implications for environmental, nutrition, human health and economic dimensions of food sustainability. Nutrients 11:E856. 10.3390/nu11040856 30995719 PMC6520741

[B18] Chopinet L. Rols M. P. (2015). Nanosecond electric pulses: A mini-review of the present state of the art. Bioelectrochemistry 103, 2–6. 10.1016/j.bioelechem.2014.07.008 25190180

[B19] Costanzo E. (2008). The influence of an electric field on the growth of soy seedlings. J. Electrostat. 66, 417–420. 10.1016/j.elstat.2008.04.002

[B20] Davalos R. V. Mir L. M. Rubinsky B. (2005). Tissue ablation with irreversible electroporation. Ann. Biomed. Eng. 33, 223–231. 10.1007/s10439-005-8981-8 15771276

[B21] Dermol J. Pakhomova O. N. Pakhomov A. G. Miklavčič D. (2016). Cell electrosensitization exists only in certain electroporation buffers. PLoS ONE 11:e0159434. 10.1371/journal.pone.0159434 27454174 PMC4959715

[B22] Eing C. Bonnet S. Pacher M. Puchta H. Frey W. (2009). Effects of nanosecond pulsed electric field exposure on *Arabidopsis thaliana*. IEEE Trans. Dielectr. Electr. Insul. 16, 1322–1328. 10.1109/TDEI.2009.5293945

[B23] Eing C. J. Goettel M. Straessner R. Gusbeth C. Frey W. (2013). Pulsed electric field treatment of microalgae—benefits for microalgae biomass processing. IEEE Trans. Plasma Sci. 41, 2901–2907. 10.1109/TPS.2013.2274805

[B24] Frey W. Strässner R. Eing C. Berghöfer T. Gusbeth C. Flickinger B. (2011). Verfahren zur Beschleunigung der Zellproliferation. European Patent No EP2308969B1. München: European Patent Office.

[B25] Gianulis E. C. Labib C. Saulis G. Novickij V. Pakhomova O. N. Pakhomov A. G. (2017). Selective susceptibility to nanosecond pulsed electric field (nsPEF) across different human cell types. Cell. Mol. Life Sci. 74, 1741–1754. 10.1007/s00018-016-2434-4 27986976 PMC7024567

[B26] Golberg A. Sack M. Teissie J. Pataro G. Pliquett U. Saulis G.. (2016). Energy-efficient biomass processing with pulsed electric fields for bioeconomy and sustainable development. Biotechnol. Biofuels 9:94. 10.1186/s13068-016-0508-z 27127539 PMC4848877

[B27] Gusbeth C. Eing C. Goettel M. Frey W. (2013). Boost of algae growth by ultra short pulsed electric field treatment, in 2013 Abstracts IEEE International Conference on Plasma Science (ICOPS) (San Francisco, CA), 1.

[B28] Haberkorn I. Buchmann L. Hiestand M. Mathys A. (2019). Continuous nanosecond pulsed electric field treatments foster the upstream performance of *Chlorella vulgaris*-based biorefinery concepts. Bioresour. Technol. 293:122029. 10.1016/j.biortech.2019.122029 31473378

[B29] Jaeger H. Knorr D. (2017). Pulsed electric fields treatment in food technology: Challenges and opportunities, in Handbook of Electroporation, ed Miklavčič D (Cham: Springer International Publishing AG), 2657–2680.

[B30] Kempkes M. (2016). Pulsed electric fields for algal extraction and predator control, in Handbook of Electroporation, ed Miklavčič D (Cham: Springer International Publishing AG, 1–16.

[B31] Kessler H. (2002). Food and Bio Process Engineering: Dairy Technology. Munich: A. Kessler.

[B32] Knoerzer K. Baumann P. Buckow R. (2012). An iterative modelling approach for improving the performance of a pulsed electric field (PEF) treatment chamber. Comput. Chem. Eng. 37, 48–63. 10.1016/j.compchemeng.2011.09.002

[B33] Kotnik T. Miklavčič D. (2006). Theoretical evaluation of voltage inducement on internal membranes of biological cells exposed to electric fields. Biophys. J. 90, 480–491. 10.1529/biophysj.105.070771 16239325 PMC1367054

[B34] Küchler A. (2009). Hochspannungstechnik. New York, NY: Springer International Publishing AG.

[B35] Lemström S. (1904). Electricity in Agriculture and Horticulture. London: The Electrician Printing & Publishing Company Ltd.

[B36] Lopez A. Rols M. P. Teissie J. (1988). 31p NMR analysis of membrane phospholipid organization in viable, reversibly electropermeabilized Chinese Hamster Ovary cells. Biochemistry 27, 1222–1228. 10.1021/bi00404a023 3365382

[B37] Mahnič-Kalamiza S. Vorobiev E. Miklavčič D. (2014). Electroporation in food processing and biorefinery. J. Membr. Biol. 247, 1279–1304. 10.1007/s00232-014-9737-x 25287023

[B38] Martínez J. M. Luengo E. Saldaña G. Álvarez I. Raso J. (2017). C-phycocyanin extraction assisted by pulsed electric field from *Artrosphira platensis*. Food Res. Int. 99, 1042–1047. 10.1016/j.foodres.2016.09.029 28865615

[B39] Mathys A. Toepfl S. Siemer C. Favre L. Benyacoub J. Hansen C. E. (2013). Pulsed Electric Field Treatment Process and Dairy Product Comprising Bioactive Molecules Obtainable by the Process. US Patent No WO2013007620A1. Washington, DC: U.S. Patent and Trademark Office.

[B40] Mattar J. R. Turk M. F. Nonus M. Lebovka N. I. El Zakhem H. Vorobiev E. (2015). *S. cerevisiae* fermentation activity after moderate pulsed electric field pre-treatments. Bioelectrochemistry 103, 92–97. 10.1016/j.bioelechem.2014.08.016 25204702

[B41] Meneses N. Jaeger H. Moritz J. Knorr D. (2011). Impact of insulator shape, flow rate and electrical parameters on inactivation of *E. coli* using a continuous co-linear PEF system. Innov. Food Sci. Emerg. Technol. 12, 6–12. 10.1016/j.ifset.2010.11.007

[B42] Miklavčič D. Mali B. Kos B. Heller R. Serša G. (2014). Electrochemotherapy: from the drawing board into medical practice. Biomed. Eng. Online 13, 1–20. 10.1186/1475-925X-13-29 24621079 PMC3995705

[B43] Ning T. Guo J. Zhang K. Li K. Zhang J. Yang Z.. (2019). Nanosecond pulsed electric fields enhanced chondrogenic potential of mesenchymal stem cells via JNK / CREB- STAT3 signaling pathway. 10:45. 10.1186/s13287-019-1133-0 30678730 PMC6346554

[B44] Pauly H. Schwan H. P. (1959). Über die Impedanz einer Suspension von kugelförmigen Teilchen mit einer Schale. Z. Naturforsch. B 14, 125–131. 10.1515/znb-1959-0213 13648651

[B45] Postma P. R. Pataro G. Capitoli M. Barbosa M. J. Wijffels R. H. Eppink M. H. M.. (2016). Selective extraction of intracellular components from the microalga *Chlorella vulgaris* by combined pulsed electric field-temperature treatment. Bioresour. Technol. 203, 80–88. 10.1016/j.biortech.2015.12.012 26722806

[B46] Raso J. Calderón M. L. Góngora M. Barbosa-Cánovas G. V. Swanson B. G. (2006). Inactivation of *Zygosaccharomyces bailii* in fruit juices by heat, high hydrostatic pressure and pulsed electric fields. J. Food Sci. 63, 1042–1044. 10.1111/j.1365-2621.1998.tb15850.x

[B47] Raso J. Frey W. Ferrari G. Pataro G. Knorr D. Teissie J. (2016). Recommendations guidelines on the key information to be reported in studies of application of PEF technology in food and biotechnological processes. Innov. Food Sci. Emerg. Technol. 37, 312–321. 10.1016/j.ifset.2016.08.003

[B48] Rego D. Redondo L. M. Geraldes V. Costa L. Navalho J. Pereira M. T. (2015). Control of predators in industrial scale microalgae cultures with pulsed electric fields. Bioelectrochemistry 103, 60–64. 10.1016/j.bioelechem.2014.08.004 25220563

[B49] Reineke K. Schottroff F. Meneses N. Knorr D. (2015). Sterilization of liquid foods by pulsed electric fields-an innovative ultra-high temperature process. Front. Microbiol. 6:400. 10.3389/fmicb.2015.00400 25999930 PMC4422003

[B50] Scherer D. Krust D. Frey W. Mueller G. Nick P. Gusbeth C. (2019). Pulsed electric field (PEF)-assisted protein recovery from *Chlorella vulgaris* is mediated by an enzymatic process after cell death. Algal Res. 41:101536 10.1016/j.algal.2019.101536

[B51] Schoenbach K. H. Joshi R. P. Kolb J. F. Chen N. Stacey M. Blackmore P. F. (2004). Ultrashort electrical pulses open a new gateway into biological cells. Proc. IEEE 92, 1122–1136. 10.1109/JPROC.2004.829009

[B52] Schoenbach K. H. Peterkin F. E. Alden R. W. Beebe S. J. (1997). The effect of pulsed electric fields on biological cells: Experiments and applications. IEEE Trans. Plasma Sci. 25, 284–292. 10.1109/27.602501

[B53] Smith K. C. Neu J. C. Krassowska W. (2004). Model of creation and evolution of stable electropores for DNA delivery. Biophys. J. 86, 2813–2826. 10.1016/S0006-3495(04)74334-9 15111399 PMC1304151

[B54] Songnuan W. Kirawanich P. (2012). Early growth effects on *Arabidopsis thaliana* by seed exposure of nanosecond pulsed electric field. J. Electrostat. 70, 445–450. 10.1016/j.elstat.2012.06.004

[B55] Steuer A. Wolff C. M. von Woedtke T. Weltmann K.-D. Kolb J. F. (2018). Cell stimulation versus cell death induced by sequential treatments with pulsed electric fields and cold atmospheric pressure plasma. PLoS ONE 13:e0204916. 10.1371/journal.pone.0204916 30312292 PMC6193580

[B56] 't Lam G. P. Postma P. R. Fernandes D. A. Timmermans R. A. H. Vermuë M. H. Barbosa M. J. (2017). Pulsed electric field for protein release of the microalgae *Chlorella vulgaris* and *Neochloris oleoabundans*. Algal Res. 24, 181–187. 10.1016/j.algal.2017.03.024

[B57] Takaki K. Kanesawa K. Yamazaki N. Mukaigawa S. Fujiwara T. Takahasi K. (1984). Application of IES pulsed power generator for mushroom cultivation. IEEE 30, 5393–5395.

[B58] Teissie J. (2017). Critical electric field and transmembrane voltage for lipid pore formation in experiments, in Handbook of Electroporation, ed. Miklavčič D (New York, NY: Springer International Publishing AG, 25–43.

[B59] Teissie J. Golzio M. Rols M. P. (2005). Mechanisms of cell membrane electropermeabilization: a minireview of our present (lack of?) knowledge. Biochim. Biophys. Acta Gen. Subj. 1724, 270–280. 10.1016/j.bbagen.2005.05.006 15951114

[B60] Toepfl S. (2011). Pulsed Electric Field food treatment - scale up from lab to industrial scale. Procedia Food Sci. 1, 776–779. 10.1016/j.profoo.2011.09.117

[B61] Toepfl S. Heinz V. Knorr D. (2005). Overview of pulsed electric field processing for food, in Emerging Technologies for Food Processing: An Overview (New York, NY: Elsevier Ltd., 69–97.

[B62] Toepfl S. Heinz V. Knorr D. (2006a). Applications of pulsed electric fields technology for the food industry, in Pulsed Electric Fields Technology for the Food Industry, eds Raso J. Heinz V (Boston, MA: Springer), 197–225. 197–221. 10.1007/978-0-387-31122-7_7

[B63] Toepfl S. Mathys A. Heinz V. Knorr D. (2006b). Review: potential of high hydrostatic pressure and pulsed electric fields for energy efficient and environmentally friendly food processing. Food Rev. Int. 22, 405–423. 10.1080/87559120600865164

[B64] Zimmermann U. Pilwat G. Riemann F. (1974). Dielectric breakdown of cell membranes. Biophys. J. 14, 881–899. 10.1016/S0006-3495(74)85956-4 4611517 PMC1334582

